# Recent advances in molecular profiling of bone and soft tissue tumors

**DOI:** 10.1007/s00256-024-04584-9

**Published:** 2024-01-17

**Authors:** D. Baumhoer, J. Hench, F. Amary

**Affiliations:** 1 Bone Tumor and DOESAK Reference Center, Institute of Medical Genetics and Pathology, University Hospital and University of Basel, Schoenbeinstrasse 40, 4031 Basel, Switzerland; 2 Institute of Medical Genetics and Pathology, University Hospital and University of Basel, Basel, Switzerland; 3 Department of Histopathology, Royal National Orthopaedic Hospital, Greater London, Stanmore, UK

**Keywords:** Methylome profiling, DNA sequencing, RNA sequencing, Bone tumors, Soft tissue tumors

## Abstract

The molecular characterization of soft tissue and bone tumors is a rapidly evolving field that has changed the perspective of how these tumors are diagnosed today. Morphology and clinico-radiological context still represent the cornerstone of diagnostic considerations but are increasingly complemented by molecular data that aid in objectifying and confirming the classification. The spectrum of analyses comprises mutation or gene fusion specific immunohistochemical antibodies, fluorescence in situ hybridization, DNA and RNA sequencing as well as CpG methylation profiling. This article provides an overview of which tools are presently available to characterize bone and soft tissue neoplasms molecularly, what limitations should be considered, and what conclusions can be drawn from the individual findings.

## Introduction

The current WHO classification of bone and soft tissue tumors lists 175 tumor subtypes, some of which are extremely rare [1]. Tumors have traditionally been grouped according to their line of differentiation and their biological behavior to guide clinical decision-making. This approach appears reasonable for lesions with obvious resemblance to normal tissues, including tumors with lipogenic, smooth muscle or osteoblastic differentiation but leaves a significant number of neoplasms in categories of uncertain differentiation. The same applies to biological behavior. Unlike many other WHO classifications, the fascicle on soft tissue and bone tumors distinguishes benign, intermediate and malignant lesions. The intermediate category comprises locally aggressive and/or rarely metastasizing tumors (<2% of cases) leaving room for subjectivity, difficulties in treatment and controversy. Aneurysmal bone cysts (ABC) for example can grow into adjacent structures and erode bone (e.g., in the craniofacial skeleton) but have been revised from locally aggressive to benign in the current classification [2]. Chondroblastomas on the other hand rarely metastasize but also have been revised to benign since metastasizing forms are exceptionally rare [3, 4].

In the last decades, yet particularly in the past few years, significant progress has been made to better understand the underlying genetic abnormalities that drive tumorigenesis. As a consequence, the so-called tumor-like lesions, for which the fourth edition of the WHO classification included an individual chapter (tumors of undefined neoplastic nature), have mostly been eliminated [5]. The criteria of neoplasia are not universally accepted but since most lesions formerly thought to represent developmentally derived disorders or hamartomas were shown to be driven by recurrent genetic events, many experts now tend to consider them as neoplastic in nature. One example is the finding of mutations in the MAP kinase signaling pathway in non-ossifying fibromas [6]. We are far from reaching an agreement on how to classify all these lesions: the current bone and soft tissue classification for example regards fibrous dysplasia (FD) as a neoplastic disease whereas the more recent classification of head and neck tumors defines FD as a “genetically based disorder of bone growth” [7, 8].

In the beginning of the 1980s, histopathology was revolutionized by the introduction of immunohistochemistry which allowed to determine and confirm lines of differentiation by protein expression detection directly on tissue sections (in situ). The last decade has been dominated by an increasing availability of techniques to characterize the molecular basis of lesions, the impact of which varies significantly among different tumor types. Bone and soft tissue tumors can be divided into four broad categories of genetic abnormalities: recurrent single nucleotide substitutions (SNV), gene rearrangements (chromosomal translocations), copy number variations (especially amplifications), and complex genomic events. For tumors in the last category like conventional osteosarcoma, only few and mostly non-specific recurrent genetic alterations have been identified so far and the diagnosis is still primarily based on morphology and clinico-radiological context. The finding of a complex genomic profile can nevertheless be supportive of a high-grade sarcoma. By contrast, several neoplasms including undifferentiated round cell sarcomas (e.g., BCOR- and CIC-related tumors) or NTRK-rearranged spindle cell tumors are mainly defined by specific molecular alterations. In the new classification of CNS tumors, a significant fraction of CNS tumors is furthermore exclusively defined by their DNA methylation profiles, even though this approach is not yet universally available in diagnostic laboratories [9, 10].

This review provides an overview of currently available approaches to characterize bone and soft tissue tumors molecularly. The advantages and limitations of different techniques are discussed and new developments are critically appraised.

## SNV and DNA sequencing

Mutation testing can be focused on a single gene, a panel of genes, the whole exome, or even the entire genome. If the differential diagnosis is narrow and testing is only performed for molecular confirmation (e.g., *GNAS* analysis in fibrous dysplasia), a single-gene approach is reasonable. Single gene tests are usually simple to establish and can be straightforward to interpret without requiring sophisticated bioinformatic expertise. However, if the expected mutation is not found, subsequent additional testing might be necessary increasing turn-around time (TAT) and costs. Independent of the method applied, the accuracy of DNA sequencing is critically dependent on the quality, integrity, and amount of nucleic acids used which can be significantly deteriorated by formalin fixation and decalcification procedures. It is therefore strongly advocated to collect and long-term preserve native tissue in a snap-frozen state from any suitable tumor sample. Additionally, rapid fixation in neutral-buffered formalin and, if required, decalcification with EDTA should be performed to achieve optimal results during molecular testing [11].

Sanger sequencing can detect changes in DNA sequences of up to 1000 bp including substitutions, insertions and deletions. It allows small amounts of input DNA but has limited sensitivity requiring a minimal variant allele fraction (VAF) of 15–20%. Due to extensive hands-on time and a comparably long TAT, the diagnostic use of Sanger sequencing continuously decreases. An alternative approach is digital droplet PCR (ddPCR) in which the DNA sample is split into nanoliter or picoliter aqueous reaction droplets within inert oil enabling detection and quantification of the target sequence in unprecedented resolution (allelic frequency down to 0.001%). Such a high sensitivity is required if the sample contains only very few cells that carry the mutation, e.g. caused by tumor heterogeneity, secondary inflammation and/or regressive changes. In fibrous dysplasia, it has been elegantly shown that the lesional cells diminish over time as a result of apoptosis [12]. Whereas *GNAS* testing is rarely needed in typical cases, it might be considered in lesions with degenerative changes which can morphologically present more ambiguous. In those cases, classical genetic testing can miss the low VAF of a *GNAS* mutation while ddPCR does not only detect but also quantifies it accurately. However, ddPCR assays must cover all pathogenic variants of interest through multiple separate probes in parallel reactions (mainly p.R201H and p.R201C but also p.Q227L amongst others for *GNAS*). This latter aspect is a general limitation of ddPCR.

In most departments, next-generation/2nd generation sequencing (NGS) with gene panels increasingly replaces single-gene testing. Particularly automated systems with integrated liquid handlers provide short hands-on and turn-around times. Molecular identifiers boost sensitivity for detecting low VAFs (thresholds currently vary between 2 and 15%) at comprehensive coverage (hundreds of loci and genes as opposed to ddPCR). Besides mutations, evidence of amplifications and gene fusions can also be provided. However, NGS requires complex bioinformatics and (at the time of writing) a mostly manual interpretation of findings. Guideline formulation is still ongoing. Additionally, rapid product cycles in sequencing chemistry and machinery negatively impact data harmonization across the globe [13–15]. This is less problematic in a diagnostic setting in which the analysis aims to identify known variants but can be challenging in a more agnostic approach focused on detecting targets for oncological treatment. With the increasingly cost-effective sequencing of whole exomes and even genomes exorbitantly more data is generated that—at some point—requires thorough evaluation. Still this non-targeted and thereby less biased comprehensive data can continuously be reevaluated for alterations in the future whenever new insights become available.

From a diagnostic standpoint, single-gene analysis or smaller gene panels currently seem sufficient to screen for most SNVs of interest in soft tissue and bone tumors. These include mutations in *CTNNB1* (= *beta-Catenin*) in desmoid-type fibromatosis, *H3-3A* in conventional giant cell tumor of bone, *H3-3B* in chondroblastoma, *IDH1/2* in cartilage tumors, *KRAS* and *FGFR1* in non-ossifying fibroma, and *GNAS* in fibrous dysplasia amongst others. Mutation-specific antibodies suitable for immunohistochemistry as surrogate markers for mutations in *CTNNB1*, *H3-3A* (p.G34W), and *H3-3B* (p.K36M) are quite reliable and specific enough to omit confirmation by sequencing [16–21]. Table 1 provides an overview of advantages and limitations of different approaches of DNA sequencing.

**Table 1:** Pros and cons of DNA sequencing techniques

Technique	Pros	Cons
Immunohistochemistry	• Low costs • Short turn-around time • Technique ubiquitously available	• Sensitivity and specificity vary significantly for individual antibodies
Sanger sequencing	• Requires little amounts of DNA • Low costs	• Low sensitivity (AF of 15-20% required) • Extensive hands-on time • Average turn-around time (2-3 days)
Digital droplet PCR	• Requires little amounts of DNA • Highest sensitivity (AF down to 0.001%) • Low costs • Short turn-around time (1 day)	• Requires separate probes for all variants of interest (multiplex approaches increasingly available)
Gene panel NGS	• Parallel sequencing of hundreds of genes in one approach • Overall high sensitivity (AF of 2-15%)	• High quality DNA required (EDTA decalcification!) • Complex bioinformatics and interpretation required • Costs vary depending on panel and technique used • Turn-around time >5 days (automated workflows can be more efficient and provide results within 48h)
Whole exome / genome sequencing	• Non-targeted approach • Evaluation of new targets at a later time possible	• Even more complex bioinformatics and interpretation required • High costs and demands for data storage

## Gene rearrangements and RNA sequencing

From the 175 soft tissue and bone tumors listed in the current WHO classification, 64 (37%) harbor recurrent gene fusions (49/117 = 42% of soft tissue tumors, 4/4 = 100% of undifferentiated small round cell sarcomas and 11/54 = 20% of bone tumors). The fusion transcripts vary significantly in type and specificity. Whereas some tumor types are characterized by highly specific gene fusions, e.g., mesenchymal chondrosarcoma (*HEY1*::*NCOA2*), others show a wider spectrum of rearrangements, some of which form fusions between members of distinct gene families, e.g., Ewing sarcoma (FET::ETS fusions) or between recurrent genes / gene family members and a variety of fusion partners, e.g., myoepithelial tumors (*EWSR1* with *POU5F1*, *PBX1*, *PBX3*, or *ZNF444*). Some fusions are associated predominantly with favorable biological behavior, e.g., *USP6*-related fusion genes were known to exclusively occur in benign neoplasms. However, the field is changing constantly and at a high pace as outlined in one of Dr. Folpe’s recent review articles “I can't keep up! (...)” [22]. Table 2 shows selected new fusions reported in the 5th edition of the WHO and beyond. Newer findings also question well-accustomed “golden rules” including the detection of *HEY1*::*NCOA2* fusions in tumors other than mesenchymal chondrosarcoma [23] and reports on rare *USP6*-rearranged cases of malignant nodular fasciitis [24, 25].

**Table 2:** Selected novel gene fusions in bone and soft tissue tumors (WHO 2020 and beyond)

Tumour type	Gene rearrangements
Acral fibromyxoid tumor	*THBS1::ADGFR2*
Angiofibroma of soft tissue	*NCOA2 (AHRR GTF2I GABI ABL1)*
Calcifying aponeurotic fibroma	*FN1::EGF*
Cellular myofibroma	*SRF::RELA*
Chondromyxoid fibroma	*GRM1*
*CIC*-rearranged sarcoma	*CIC::DUX4 (FOXO4, LEUTX, NUTM1, NUTM2A0*
Desmoplastic fibroblastoma	*FOSL1*
Epithelioid fibrous histiocytoma / dermatofibroma	*ALK*
Epithelioid hemangioma	*FOS FOSB*
*EWSR1::SMAD3*-positive fibroblastic tumor	*EWSR1::SMAD3*
Keratin-positive giant cell tumor / xanthogranulomatous epithelial tumor of soft tissue	*HMGA2::NCOR2*
*GLI1-*rearranged tumors	*GLI1*
Glomus tumor	*NOTCH* gene family
Hyalinizing epithelioid tumor of the hand	*ORG::FOXO3 FOXO4*
Hybrid nerve sheath tumor (schwannoma / perineurioma)	*CHD7::VGLL3*
*ICA1L::SRF* fusion tumor	*ICA1L::SRF*
Intra-osseous spindle cell rhabdomyosarcoma	*TFCP2::NCOA2*
*KMT2A*-rearranged sarcoma	*KMT2A (YAP1 PRRX)*
Lipofibromatosis	receptor tyrosine kinases
Mixed tumor / Myoepithelioma of soft tissue	*EWSR1 FUS PLAG1*
Myxoid fibroblastic tumor of the vocal cord (inflammatory myofibroblastic tumor)	*TIMP3::ALK*
*NTRK*-rearranged spindle cell neoplasm	*NTRK1 NTRK2 NTRK3*
Osteoblastoma	*FOS* (*FOSB* in exceptionally rare cases)
Osteoid osteoma	*FOS*
Phosphaturic mesenchymal tumor	*FN1::FGFR1*
*PRRX::NCOA1/2*-rearranged fibroblastic tumor	*PRRX::NCOA1/2*
Round cell sarcoma with EWSR1-non-ETS fusion	*EWSR1::NFATC2, FUS::NFATC2, EWSR1::PATZ1*
Sarcoma with *BCOR* genetic alterations	*BCOR* rearrangements (*BCOR::CCNNB3*)
Simple bone cyst	*EWSR1::NFATC2*
Soft tissue chondroma	*FN1 (FGFR1 FGFR2 other partners)*
Solitary fibrous tumor	*NAB2::STAT6*
Superficial CD-34 positive fibroblastic tumor	*PRDM10*
Synovial chondromatosis	*FN1 ACVR2A*

**Fig. 1:**
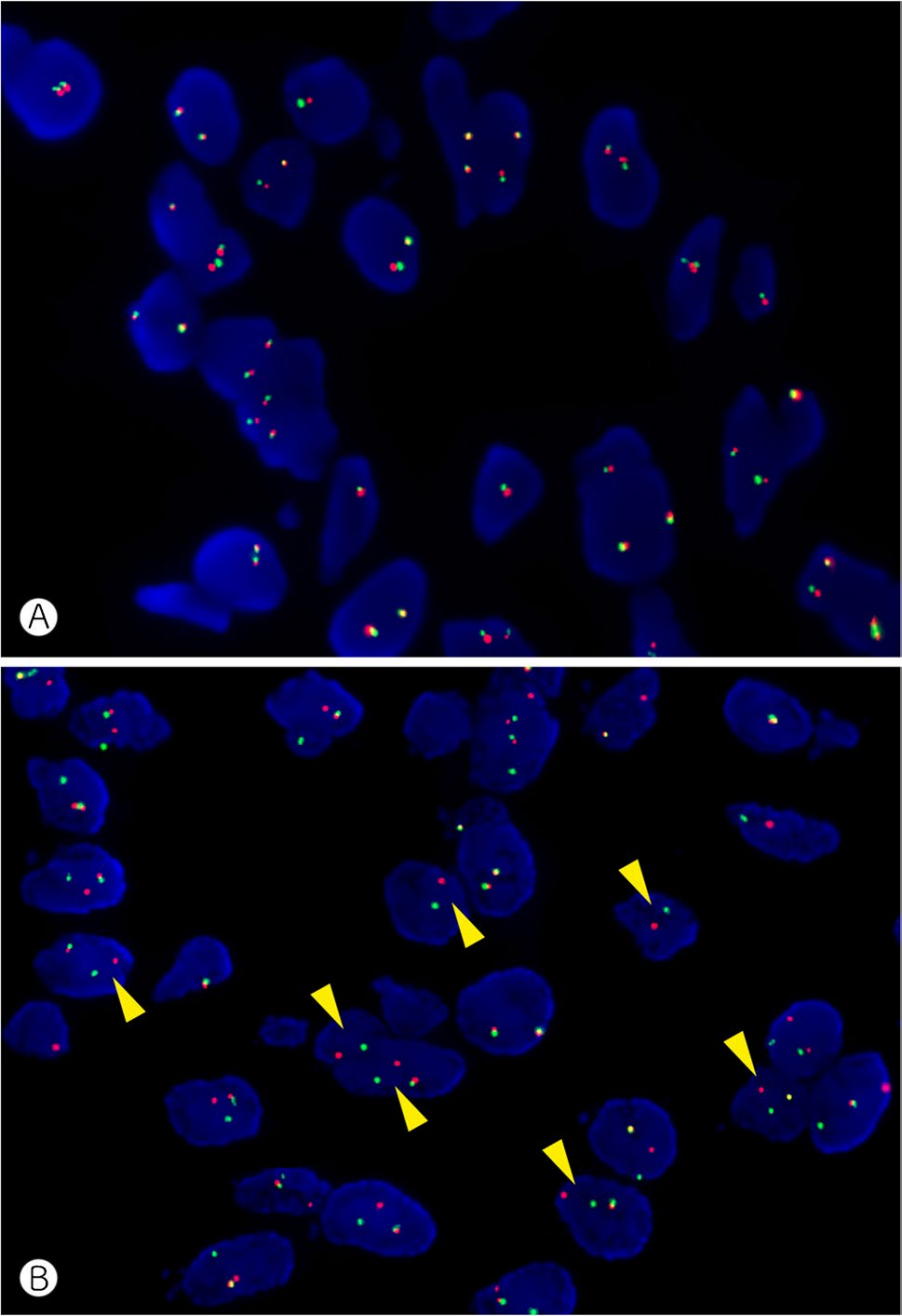
*USP6* FISH analysis in a wild-type (**A**) and rearranged tumor (**B**). The yellow arrowheads show the spatial separation of green and red hybridization signals

For the detection of gene fusions, fluorescence in-situ hybridization (FISH) is an established and easy-to-use method in many pathology laboratories. Usually, hybridization probes flanking a gene of interest demonstrate that the normal DNA sequence of a gene has been disrupted, providing indirect evidence of a rearrangement. In a wild-type configuration, the dual-color break-apart probes lie in close proximity to each other, generating a single, merged-color signal (e.g., green & red = orange). If spatially separated, the probes light up as individual signals (green apart from red), demonstrating a chromosomal break between the two investigated genomic locations (Fig. 1); the partner gene involved in a potential gene fusion remains unknown. FISH is known to miss some rearrangements, e.g., intrachromosomal fusions such as *EWSR1*::*PATZ1* in which the spatial resolution is insufficient to differentiate normal and aberrant patterns of the hybridization signals [26]. As a workaround, a dual fusion FISH design targets both potential fusion partners. In any case, FISH analysis is DNA-based and therefore provides no information about the transcription or functional integrity of a rearrangement. Due to the large intron size typically flanking chromosomal breaks, usually exceeding the average DNA fragment length requirements in DNA sequencing approaches, mRNA sequencing, typically through PCR-amplified cDNA after reverse transcription, is the mainstay of gene fusion diagnostics. This latter technique benefits from a high sensitivity since the majority of functionally relevant gene fusions are overexpressed.

**Fig. 2:**
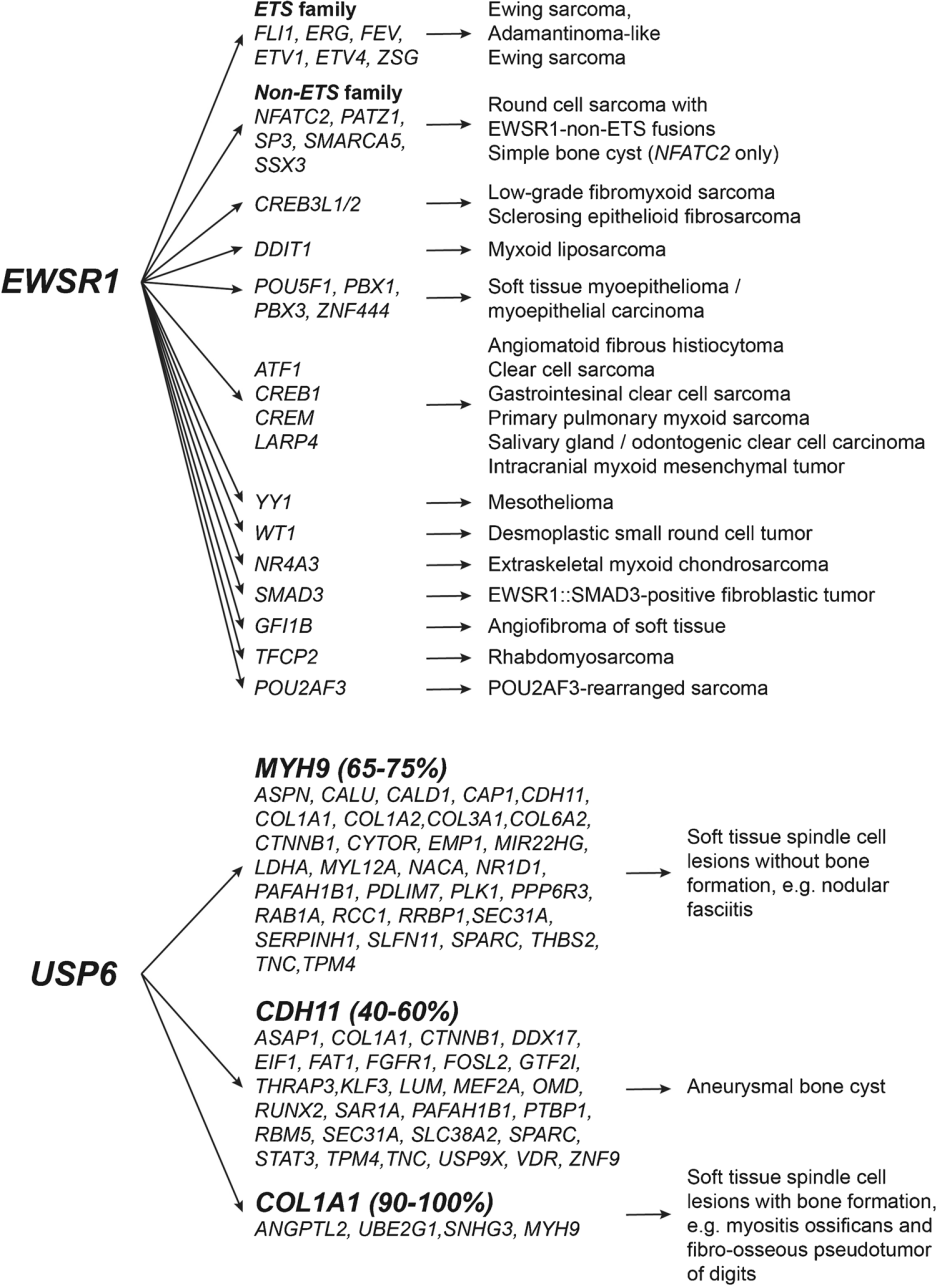
The spectrum of *EWSR1*- and *USP6*- rearranged tumors (modified from [27, 28])

For single gene fusion tests, rtPCR can be used requiring a specific set of primers covering both fusion partners. The same (amplicon-based) approach can be used in NGS panels, e.g., as an expression imbalance assay, but the limitation to only identify predefined gene fusions / breakpoints remains. Today, the most commonly used assays require only one of the fusion partners to be recognized by a specific primer set, while a second universal primer binds to a sequence on an adapter downstream of the fusion partner. This enables the detection also of novel partner genes and provides information on the breakpoints / exons involved, whether the fusion is in-frame, and the level of expression and is particularly helpful for rearrangements involving genes as *USP6* or *EWSR1* which are known to form fusion transcripts with multiple partner genes (Fig. 2). The limitation, that one of the fusion partner genes must be covered by the primer set, remains. Whole transcriptome sequencing is therefore likely to replace panel sequencing in the future as soon as the prices—particularly also for data storage and computational analysis—drop below the threshold currently set by targeted protocols.

**Fig. 3:**
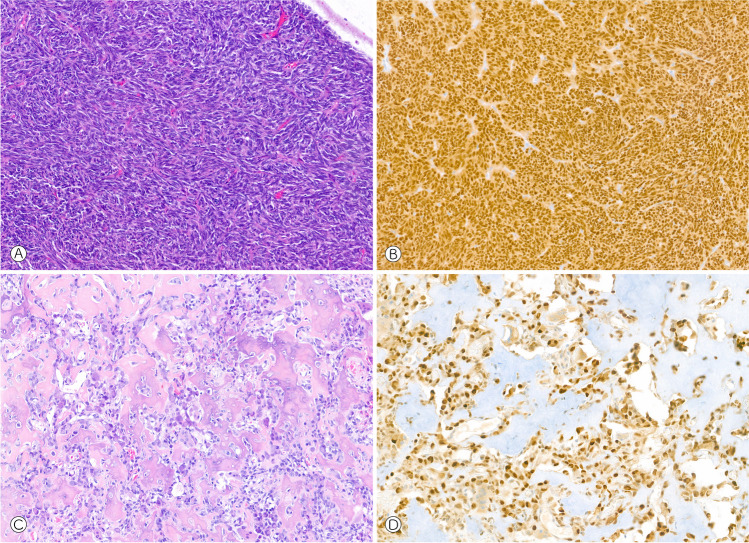
Synovial sarcoma showing consistent nuclear positivity in an immunohistochemical staining with the fusion-specific antibody SS18-SSX (**A**: H&E, 100×, **B**: immunohistochemistry, 100×). Osteoblastoma revealing strong nuclear expression of FOS (**C**: H&E, 100×, **D**: immunohistochemistry, 150×)

A major drawback for all sequencing approaches is the highly variable, commonly poor, and constantly decreasing RNA quality in FFPE samples, particularly following decalcification. Studies have shown that up to 50% of archived FFPE samples may not pass the pre-sequencing quality controls [29]. To avoid false negative results, native material should be collected from any (neoplastic) biopsy and resection specimen whenever possible and transferred to long-term storage in a snap-frozen state. A smart alternative to FISH and RNA sequencing is immunohistochemistry against surrogate markers for gene fusions. *FOS* rearrangements in osteoid osteoma and osteoblastoma for example lead to an overexpression of the FOS protein that can be detected immunohistochemically [30]. For other rearrangements, fusion-specific antibodies are available, e.g., for the SS18-SSX fusion in synovial sarcoma [31] (Fig. 3). These tests are easily implemented, affordable, fast, and less demanding with respect to tissue preservation.

## Methylome profiling and copy number analysis

Histopathologic assessment of tissue specimens is based on pattern recognition. The methylation status of CpG sites, of which—putatively—around 30 million are distributed throughout the genome, forms another pattern that correlates with cellular differentiation and can be used to epigenetically classify cell types, tissues and neoplasms. CpG sites are DNA sequences in which a cytosine is followed by a guanine and the cytosine residue can be either methylated or not. Commonly used assays (e.g., Infinium MethylationEPIC, Illumina, USA) interrogate around 900'000 of those CpG sites and well-preserved FFPE samples are usually sufficient to provide evaluable data. To recognize a tumor type by its methylome, individual methylation classes have to be established for which generally 8–12 representative cases are required per entity. These classes serve as a ground truth against which new (and unknown) tumor samples are then compared with using machine learning algorithms.

The first methylation classifiers that found their way into clinical routine use and WHO classification have been developed for brain tumors and proved to be highly reliable and accurate [32]. The new WHO classification for CNS tumors even includes new tumor types that have been exclusively defined by their methylome profiles [9]. The same group of neuropathologists who established the first brain tumor classifier meanwhile also published a sarcoma classifier and several other groups have validated this classifier with independent and well-characterized series of soft tissue and bone tumors [33–35]. The sensitivity and specificity of individual tumor classes vary with fusion driven neoplasms generally forming more distinct clusters and less well (molecularly) defined lesions, including MPNST and clear cell chondrosarcoma, displaying more ambiguous results. The classifier uses a supervised ML approach (random forest) and provides a confidentiality score for predicting its accuracy (https://www.molecularneuropathology.org/mnp/). Another platform, based on unsupervised ML and available at no cost can be found at www.epidip.org.

**Fig. 4:**
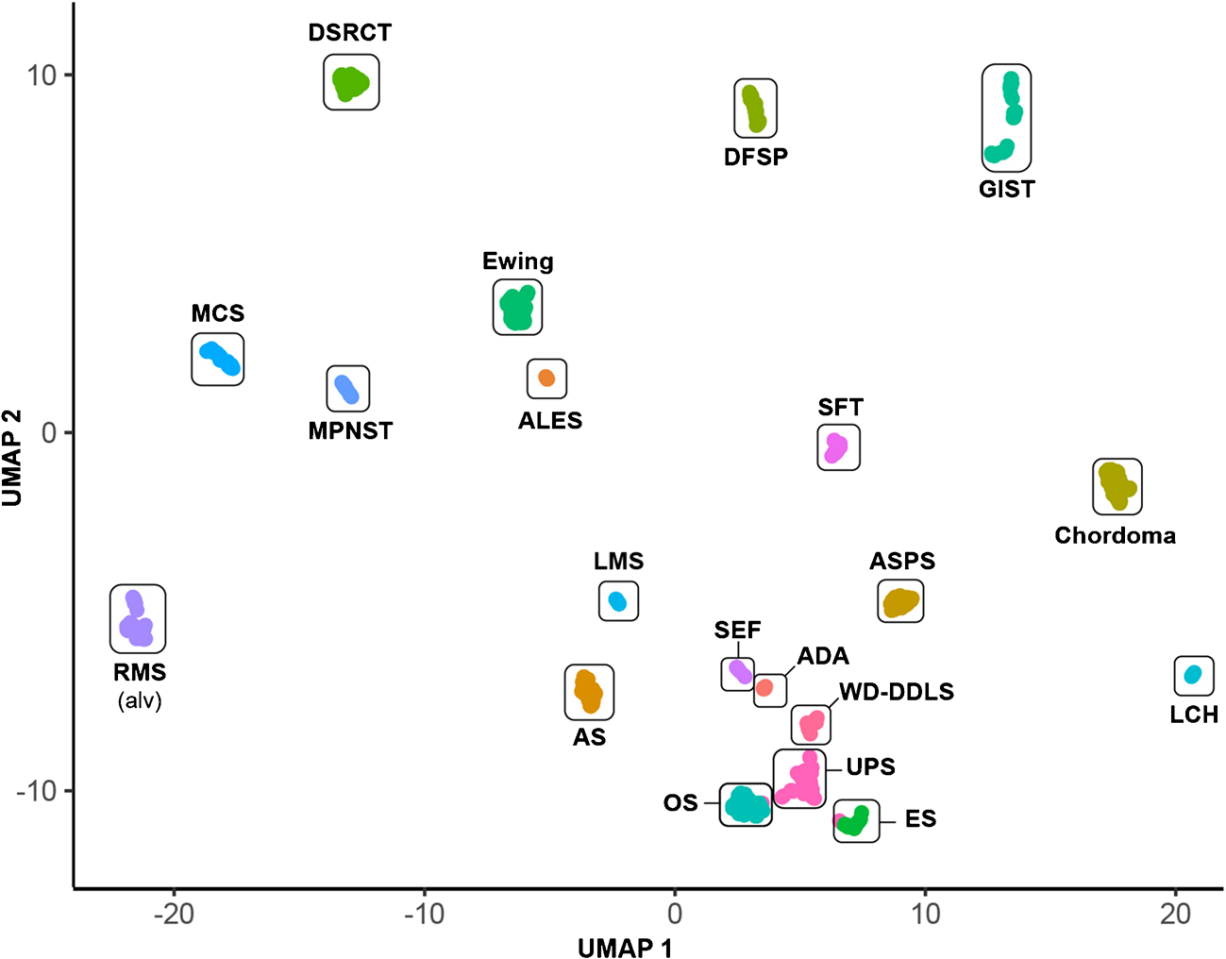
DNA methylation-clustering of selected malignant bone and soft tissue tumors. Uniform Manifold Approximation and Projection (UMAP) analysis of internal and publicly available methylomes (*n*=700) assessed by genome-wide DNA methylation arrays (Illumina BeadChip 450K or EPIC). Clustering was performed on the top 25’000 most variably methylated probes. (Abbreviations: conventional adamantinoma (ADA, *n*=9); adamantinoma-like Ewing sarcoma (ALES, *n*=9); angiosarcoma (AS, *n*=37); alveolar soft part sarcoma (ASPS, *n*=35); chordoma (*n*=50); dermatofibrosarcoma protuberans (DFSP, *n*=44); desmoplastic small round cell tumor (DSRCT, *n*=40); epithelioid sarcoma (ES, *n*=25); Ewing sarcoma (*n*=50); gastrointestinal stromal tumor (GIST, *n*=50); conventional osteosarcoma (OS, *n*=50); Langerhans cell histiocytosis (LCH, *n*=12); leiomyosarcoma (LMS, *n*=17); mesenchymal chondrosarcoma (MCS, *n*=39); malignant peripheral nerve stealth tumor (MPNST, *n*=25); alveolar rhabdomyosarcoma (RMS alv, *n*=50); sclerosing epithelioid fibrosarcoma (SEF, *n*=14); solitary fibrous tumor (SFT, *n*=24); undifferentiated pleomorphic sarcoma (UPS, *n*=49); well-/dedifferentiated liposarcoma (WD-DDLS, *n*=21)

Methylome classifiers can only recognize lesions of which methylation classes have been established in the underlying ground truth dataset. The sarcoma classifier from Heidelberg so far includes only 38/117 (32%) soft tissue, 3/4 (75%) undifferentiated small round cell sarcomas and 14/54 (24%) bone tumors, adding up to 52/175 (30%) soft tissue and bone tumors included in the current WHO classification [33]. Some methylation classes have been generated by only few representative tumor samples which might further weaken the diagnostic accuracy of the classifier. To fully appraise the diagnostic potential of methylome classifiers for soft tissue and bone tumors, a platform would need to include reliable methylation classes based on a solid ground truth for all 175 tumor types. This endeavor would benefit from an international collaborative approach and has not been completed yet (Fig. 4).

As published only recently for brain tumors, methylome profiling using ultra-fast sequencing techniques can provide an accurate classification of tumors within a few hours [36–38]. One method increasingly applied is nanopore (3rd generation parallel) sequencing in which single DNA molecules (without prior amplification) are electrically pulled through transmembrane proteins (= nanopores) embedded in a nonconductive membrane. In contrast to targeted sequencing, this technique analyzes what randomly passes through the pores and the coverage (including a direct measurement of methylated CpG sites) increases with time. After exceeding an arbitrarily defined cut-off of data density, the sequencing is stopped. Despite a lower resolution compared to EPIC arrays, the data is usually sufficient to reach a reliable prediction, under optimal circumstances in less than 3 h. The sole limitation to nanopore sequencing is the dependence on native (or alcohol-preserved) tissue specimens since formalin fixation breaks the DNA strands and precludes this approach.

**Fig. 5:**
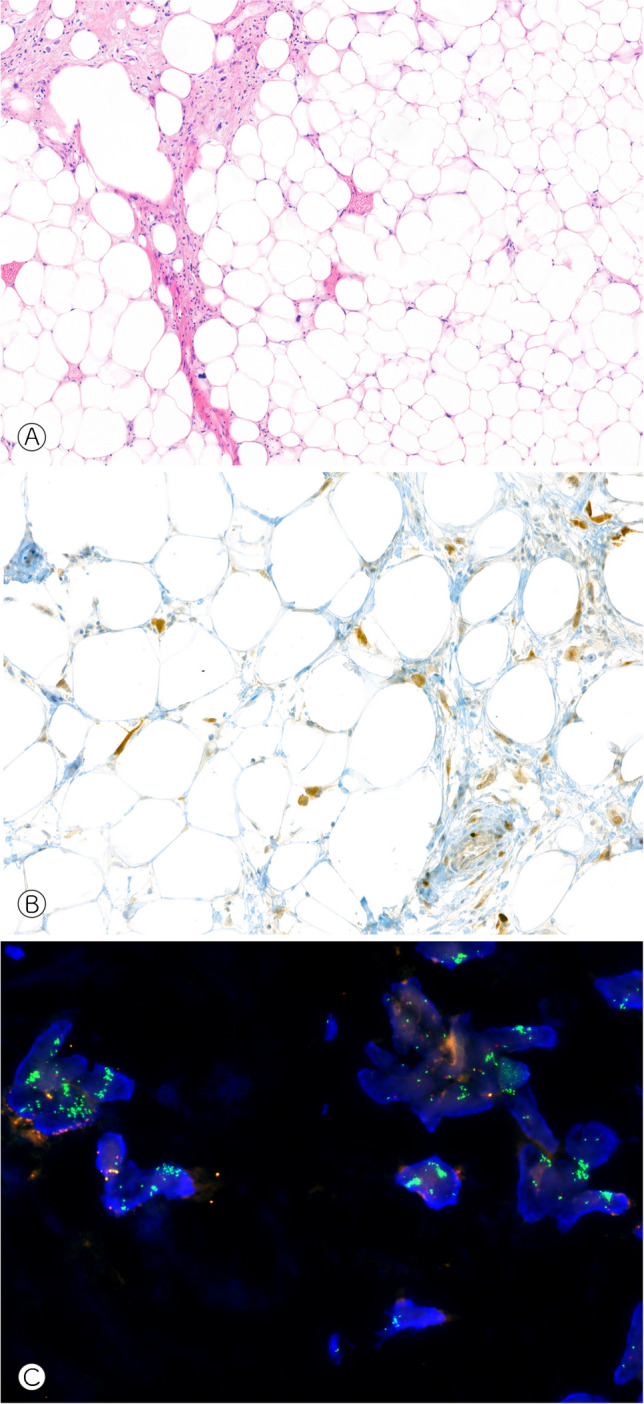
Atypical lipomatous tumor / well-differentiated liposarcoma showing mostly mature appearing multilobulated fatty tissue with atypical adipocytic cells and intermingled lipoblasts (**A**, H&E, 75×). Immunostaining against MDM2 reveals nuclear positivity of intermingled atypical cells (**B**, 150×) and FISH analysis shows clouds of amplified MDM2 hybridization signals (in green, **C**).

**Fig. 6:**
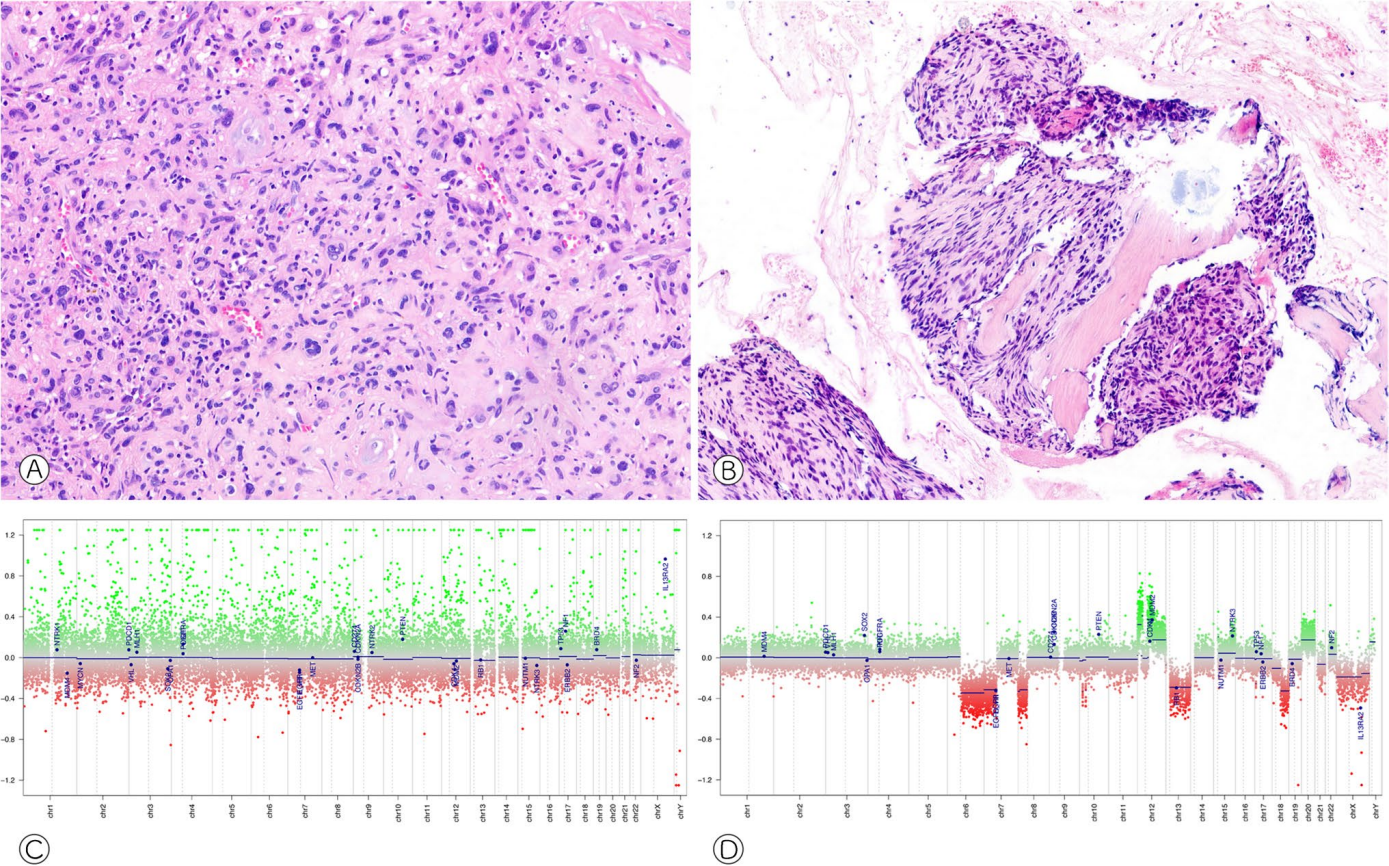
Recurrent giant cell tumors of bone with corresponding copy number profiles. Case #1 shows symplastic atypia (**A**, H&E, 150×) and a flat copy number profile (**C**). Case #2 reveals moderately atypical spindle cells encasing preexisting trabeculae indicating osteodestructive growth (**B**, H&E, 150×). The copy number profile demonstrates multiple chromosomal gains and losses, in keeping with malignant transformation

**Fig. 7:**
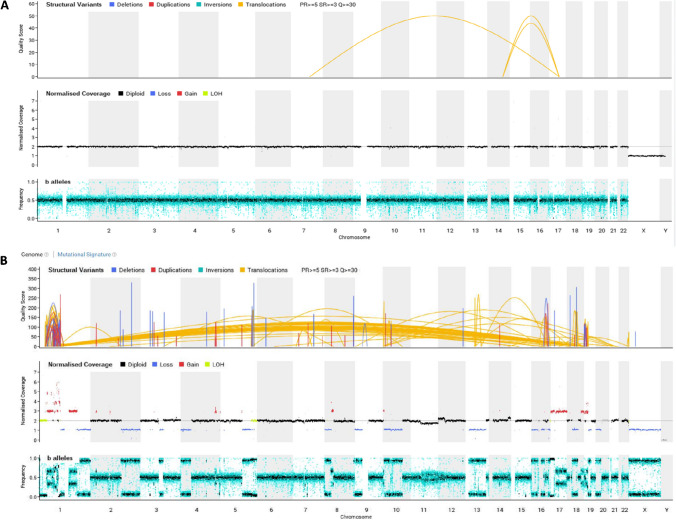
WGS plots (CNVs, coverage and B-allele frequency) showing a quiet and flat genome of an osteoblastoma (**A**) with a rearrangement involving FOS on chromosome 14; compared to a plot of a high-grade osteosarcoma (**B**) with complex abnormalities including many chromosomal gains and losses

As another layer of diagnostically meaningful information, copy number variations (CNV) can be derived from high-dimensional CpG methylome profiles, from both microarrays and nanopore, the latter having a lower resolution. Microarray data can be helpful in detecting amplifications or deletions of single genes / smaller stretches of DNA including several genes. *MDM2* amplifications for example are the diagnostic hallmark of well-differentiated / dedifferentiated liposarcoma but also occur in parosteal (>85%) and low-grade central (25-30%) osteosarcoma as well as in intimal sarcoma [39, 40]. Immunohistochemistry can be helpful as a surrogate marker but due to lack of specificity (histiocytes and multinucleated giant cells are usually positive as well) should generally be confirmed by FISH, particularly in the initial biopsy (Fig. 5). *Rb1* deletions are typically present in a variety of soft tissue neoplasms including spindle cell / pleomorphic lipoma, atypical spindle cell / pleomorphic lipomatous tumor, pleomorphic liposarcoma, myofibroblastoma, cellular angiofibroma, and acral fibromyxoma [41]. CNV profiles furthermore tend to correlate with biological behavior. Whereas benign lesions (with the exception of some fusion-driven tumors) usually lack copy number alterations, high-grade sarcomas often show extensive chromosomal gains and losses. In difficult cases, where the fundamental question lies between a benign or malignant entity, such as the classic conundrum between an osteoblastoma and osteosarcoma or between a giant cell tumor with symplastic / regressive changes and a malignant giant cell tumor, whole genome sequencing or copy number plots generated from DNA panels provide an additional layer of safety when it shows a flat profile (favoring benign disease) or a complex array of abnormalities (more supportive of a malignant tumor [42] (Figs. 6–7). Distinction of complex aberrations from flat profiles is easily possible also with fast-track nanopore sequencing.

## What defines a neoplasm and how should this impact the nomenclature?

The first WHO classification of bone tumors from 1972 was exclusively based on histological criteria, particularly on cellular differentiation and matrix formation. For the less differentiated neoplasms without intercellular material, the prediction of biological behavior guided subtyping. It was underlined already at this time, that an interdisciplinary approach including clinical, radiological, and histopathological features, supplemented by biochemical and hematological studies, was mandatory to accurately classify bone tumors. Immunophenotyping specified and objectified tumor subtyping but was introduced systematically only in the third edition of the WHO classification published in 2002. The nomenclature was refined over time but kept relatively stable.

The molecular characterization confirmed the majority of established tumor types and helped to refine the morphological assessment. Some tumor types show highly specific alterations like synovial sarcoma (*SS18*::*SSX* fusions) and chondroblastoma (*H3-3B* mutations), other mutations are found in tumor subgroups such as *IDH1/2* mutations in cartilage neoplasms. Some mutations widened the spectrum of tumor types, like *H3-3A* in conventional giant cell tumor of bone. Since the mutation was also found in fibrous histiocytoma of bone, this lesion is no longer considered a separate entity and is now perceived as a variant of giant cell tumor without giant cells. Similarly, giant cell lesions of the small tubular bones are now considered “solid” aneurysmal bone cysts (ABC) since the majority show rearrangements of *USP6* which can be identified in an almost uniformly benign group of formerly thought to be unrelated lesions (ABC, myositis ossificans, nodular fasciitis, cranial fasciitis, fibroma of tendon sheath, and fibro-osseous pseudotumor of the digits, Fig. 2). These findings challenge the established and rather descriptive nomenclature of both tumor types. Likewise, similar joinings of morphologically distinct patterns into a single molecular entity was also observed in some brain tumors (WHO 2021, spindle cell oncocytoma / granular cell tumor of the sellar region / pituicytoma), which are now considered a single entity with multiple, mostly irrelevant, morphological patterns [9].

The increasing availability of fusion testing resulted in a surge of newly reported rearrangements of unknown pathogenicity and specificity. Some tumors are defined by gene fusions despite the lack of uniform histologic criteria, e.g., *NTRK*-rearranged spindle cell tumors. Methylome profiling on the other hand showed conventional chondrosarcomas to form 4–5 molecular subgroups that cannot be distinguished histologically. Is an *H3-3A* mutation or an *MDM2* amplification detected in a conventional high-grade osteosarcoma, furthermore, sufficient to suggest a malignant giant cell tumor or a low-grade central osteosarcoma with high-grade transformation [43]?

Until more evidence becomes available, the focus of tumor subtyping should remain on clinical utility to guide decision-making. The nomenclature of soft tissue and bone tumors will remain a matter of debate but should be revised only after thorough consideration to avoid confusion among clinicians. The WHO classifications have always been based on extensive literature review and scientifically sound and convincing data which must remain the foundation also for future amendments.

## Outlook

The increasingly available plethora of molecular techniques has substantially changed the way bone and soft tissue tumors are characterized and diagnosed. Whereas morphology and immunophenotyping are still the backbone to classify neoplastic disease, characteristic mutations, fusion transcripts, CpG methylome profiles, and whole exome/genome sequencing can help to objectify and confirm the diagnosis. It is beyond the scope of this article to cover all available methods and it is difficult to predict how we will diagnose bone and soft tissue tumors ten or twenty years from now. If methylome profiling turns out to be as reliable as in brain tumors, this technique might have a substantial impact, particularly if supported by ultra-fast technologies like nanopore sequencing. Whereas RNA panel sequencing can easily take 2–3 weeks and in case of a negative result might need to be complemented by additional tests, a molecular CpG methylome profile including CNV within few hours could significantly speed up clinical decision making. Multiplex immunophenotyping, proteomics, spatial transcriptomics, and single-cell sequencing could shed more light on the molecular pathogenesis of tumors and identify new targets for diagnostic or even therapeutic purposes. Other promising avenues comprise generative AI and large language models that will analyze histologic (and radiologic) images along with associated clinical data at an unprecedented precision.

The amount of data generated by genomic sequencing today is greater than the available targets for treatment and clinical trials opened for sarcomas. Although current patients may not yet benefit directly, this data, potentially along methylation profiling, might help to better stratify patients and tumor subtypes that differ in clinical behavior despite a seemingly identical histology. Hopefully, this progress can be translated also into novel treatment modalities resulting in better patient care and outcomes soon.
